# Electroacupuncture Protects Against Post‐MI Heart Failure Through Autonomic Regulation and α7nAChR Activation

**DOI:** 10.1155/crp/9919619

**Published:** 2026-06-22

**Authors:** Weiting Wang, Jiaojiao Wu, Yueling Xu, Qiao Lin, Ke Wang, Aijun Liu, Jia Zhou

**Affiliations:** ^1^ Acupuncture Anaesthesia Clinical Research Institute, Yueyang Hospital of Integrated Traditional Chinese and Western Medicine, Shanghai University of Traditional Chinese Medicine, Shanghai, China, shutcm.edu.cn; ^2^ Shanghai Acupuncture Clinical Research Center, Yueyang Hospital of Integrated Traditional Chinese and Western Medicine, Shanghai University of Traditional Chinese Medicine, Shanghai, China, shutcm.edu.cn; ^3^ Department of Pharmacy Research, Yueyang Hospital of Integrated Traditional Chinese and Western Medicine, Shanghai University of Traditional Chinese Medicine, Shanghai, China, shutcm.edu.cn

**Keywords:** α7nAChR, electroacupuncture, heart failure, Neiguan acupoint, vagal nerve, ventricular remodeling

## Abstract

**Objective:**

This study investigated whether electroacupuncture (EA) at Neiguan (PC6) acupoint alleviates adverse myocardial remodeling in post–myocardial infarction (MI) heart failure (HF) by activating the vagus nerve–mediated cholinergic pathway and its downstream α7nAChR‐Akt‐HIF‐1α‐VEGF signaling cascade.

**Methods:**

Male Sprague Dawley (SD) rats were randomized into sham, MI model, and EA groups (*n* = 6 per group). MI was induced by ligation of the left anterior descending coronary artery. EA was applied at PC6 for 14 days starting 1 week after MI induction. Cardiac function and structure were evaluated using echocardiography and histological examination. The expression levels of choline acetyltransferase (ChAT), α7‐nicotinic acetylcholine receptor (α7nAChR), phosphorylated Akt (*p*‐Akt), hypoxia‐inducible factor‐1α (HIF‐1α), and vascular endothelial growth factor (VEGF) were analyzed by real‐time quantitative PCR, Western blotting, and immunohistochemistry.

**Results:**

EA intervention significantly attenuated pathological myocardial remodeling and improved cardiac function. Compared with the model group, EA treatment markedly enhanced cardiac function, increased contractility, reduced ventricular dilation, restored autonomic balance, and alleviated myocardial necrosis and fibrosis. Molecular studies revealed that EA treatment increased the expression of ChAT and α7nAChR, elevated *p*‐Akt levels, and upregulated the expression of HIF‐1α and VEGF. These findings suggest the activation of the vagus nerve–cholinergic signaling pathway and its downstream prosurvival and pro‐angiogenic pathways.

**Conclusions:**

EA attenuated maladaptive ventricular remodeling and improved cardiac performance. These effects were associated with modulation of autonomic function and engagement of the α7nAChR/Akt/HIF‐1α/VEGF signaling axis, supporting a potential neuro‐immune‐angiogenic mechanism underlying EA‐mediated myocardial repair during the subacute post‐MI phase.

## 1. Introduction

Myocardial infarction (MI) is a major precursor of heart failure (HF), which remains a leading global cause of morbidity and mortality [1]. Cardiovascular function is tightly regulated by the autonomic nervous system (ANS) through the balance of sympathetic and parasympathetic activity [2]. Post‐MI ANS dysregulation, characterized by excessive sympathetic activation and diminished vagal activity, directly contributes to and interacts with multiple pathophysiological mechanisms [3]. These mechanisms include neuroendocrine activation, inflammatory response, oxidative stress, apoptosis, extracellular matrix (ECM) remodeling, and calcium homeostasis disruption [4]. Collectively, these factors trigger the onset and progression of pathological ventricular remodeling, ultimately leading to severe clinical outcomes of HF [2, 5]. Although timely reperfusion and guideline‐directed medical therapy (GDMT) improve early survival, they do not prevent long‐term adverse remodeling [6, 7]. Reversing or attenuating ventricular remodeling therefore represents a key therapeutic goal for post‐MI HF [8]. Identifying comprehensive strategies that modulate autonomic tone, promote myocardial repair, limit adverse remodeling, and improve long‐term prognosis is a pressing need in cardiovascular medicine [9].

Vagus nerve stimulation (VNS) has demonstrated cardioprotective effects in numerous experimental studies [10–12]. Preclinical and early clinical data suggest that VNS may serve as an adjunctive therapy in chronic heart failure (CHF) [13]. These effects are largely attributed to the cholinergic anti‐inflammatory pathway (CAP), mediated by ACh released from the vagus nerve acting on α7‐nicotinic acetylcholine receptors (α7nAChR) [14–16]. Electroacupuncture (EA) is a combination of traditional acupuncture and electrical stimulation, and its advantages include the fact that the stimulus can be controlled and repeated objectively [17]. In recent years, EA has been proposed as a noninvasive alternative or adjunctive approach to VNS [18], demonstrating potential cardioprotective effects, particularly when applied to Neiguan (PC6) acupoint on the pericardium meridian [19–22]. EA at PC6 has been shown to modulate cardiac autonomic nervous activity and neuroendocrine imbalance [21], attenuate inflammation and oxidative stress, and reduce signaling that drives adverse postinjury remodeling [19]. Small‐scale human studies further demonstrated that EA enhances heart rate variability (HRV), consistent with augmented vagal activity [22]. Experimental research indicates that EA enhances vagal activity and exerts anti‐inflammatory effects through cholinergic pathways, particularly those involving α7nAChR [23].

However, the pathogenesis of post‐MI HF extends beyond inflammation, involving maladaptive ventricular remodeling, microcirculatory dysfunction, and cardiomyocyte apoptosis/necrosis [24, 25]. Whether EA‐mediated activation of α7nAChR contributes to cardioprotection in these broader processes remains unclear. Therefore, this study aimed to investigate the role of EA‐induced α7nAChR signaling in attenuating adverse remodeling during the transition from MI to HF, and to explore its potential as a safe, nonpharmacological neuromodulatory strategy.

## 2. Methods

### 2.1. Animals

SPF male Sprague Dawley (SD) rats (180–200 g) were provided by Shanghai Silek Experimental Animal Co., Ltd. Rats were housed in individually ventilated cages (IVCs) lined with autoclaved corn cob bedding, maintained at 24 ± 1°C, 50%–70% humidity, and a 12/12‐h light/dark cycle (lights on at 08:00), with free access to tap water and standard rat chow.

### 2.2. Induction of MI and Grouping

MI was induced by permanent ligation of the left anterior descending artery (LAD) under isoflurane anesthesia. A left thoracotomy was performed at the fourth intercostal space to expose the heart, and the LAD was ligated approximately 1–2 mm from its origin with a 7‐0 silk suture. Successful ligation was confirmed by immediate pallor of the anterior ventricular wall and ST‐segment elevation (≥ 0.2 mV) on electrocardiogram (ECG). The thoracic cavity was closed after expelling residual air, and rats were allowed to resume spontaneous breathing. Sham‐operated rats underwent the same procedure without LAD ligation. The perioperative mortality rate was ∼30%, mostly within the first week. One week after surgery, surviving rats were randomly divided into a model group (*n* = 6) and an EA treatment group (*n* = 6).

### 2.3. Time Window

Post‐AMI Day 7 corresponds to the transition from the inflammatory to the proliferation/early maturation phase of healing. At this stage, intervention can influence macrophage polarization, fibroblast activity, and ECM deposition, thereby affecting subsequent scar formation and ventricular geometry changes. By Day 21, early structural and functional remodeling becomes apparent, including fibrosis severity, left ventricular ejection fraction (LVEF), and ventricular volumes.

### 2.4. EA Intervention

Seven days after LAD induction, rats in the EA group were placed in a supine position under 3% isoflurane for induction and 1.5% for maintenance. Bilateral PC6, located between the tendons of the palmaris longus and flexor carpi radialis muscles, approximately 3 mm proximal to the transverse wrist crease, were selected for stimulation. Sterile stainless steel acupuncture needles (0.25 mm diameter) were inserted perpendicularly to a depth of 2–3 mm and connected to an EA apparatus (SDZ‐V, HWATO, Suzhou Medical Appliance Factory, Suzhou, China). A growing body of research has demonstrated that EA stimulation with a 2 Hz continuous wave exerts a significant protective effect on myocardial tissue against myocardial injury [17, 26]. Based on these findings, a continuous 2 Hz waveform was delivered at a current intensity of 2 mA. Each EA session lasted 30 min and was performed once daily for 14 consecutive days. Rats in the sham and model groups were handled in the same way but received neither needle insertion nor electrical stimulation.

### 2.5. Echocardiography (ECHO)

ECHO was performed to assess cardiac function after 14 days of EA intervention. Transthoracic ECHO was conducted under light isoflurane anesthesia (1.5% isoflurane), with anesthesia maintained to ensure stable hemodynamics throughout the examination without significant interference with cardiac function. A high‐frequency (HF) ultrasound system (Vevo 2100 system, VisualSonics, Canada) was used for image acquisition, and standard two‐dimensional (2D) echocardiographic images (including parasternal long‐axis views and papillary muscle‐level parasternal short‐axis views) were obtained prior to recording M‐mode tracings. From M‐mode tracings, key left ventricular parameters were calculated, including left ventricular internal diameter at end‐diastole (LVIDd) and end‐systole (LVIDs), fractional shortening (FS), and ejection fraction (EF), with EF and FS serving as primary indicators of global left ventricular systolic function. All measurements were averaged over three consecutive, stable cardiac cycles and analyzed offline by an investigator who was blinded to group allocation to minimize subjective bias and ensure the accuracy and reliability of the results.

### 2.6. HRV

HRV was assessed in conscious, unrestrained rats 14 days after EA treatment. Following acclimatization to the testing environment, surface ECG signals were continuously acquired for a minimum of 30 min under quiet resting conditions using a dedicated data acquisition system. Frequency‐domain analysis of stable signal segments was performed using LabChart software, including low‐frequency power (LF), HF power, and LF/HF ratio. These parameters were quantified to characterize vagal (parasympathetic) modulation, combined sympathovagal inputs, and overall autonomic balance, respectively, thereby evaluating the regulatory effects of EA on cardiac ANS function.

### 2.7. Histopathological Staining

At the study endpoint, all rats were euthanized via intraperitoneal injection of an overdose of pentobarbital sodium (150 mg/kg, Sinopharm Chemical Reagent Co., Ltd., China). Then, rat hearts were excised, rinsed in cold PBS, and fixed in 4% paraformaldehyde. Paraffin‐embedded sections (5 μm) were subjected to hematoxylin–eosin (HE), Masson’s trichrome, and wheat germ agglutinin (WGA) staining, which were used to evaluate general myocardial morphology (including inflammation and necrosis), myocardial fibrosis, and cardiomyocyte hypertrophy (via cross‐sectional area measurement), respectively. Vascular endothelial growth factor (VEGF) expression was assessed by immunohistochemistry. Image analysis was performed using ImageJ software by an investigator blinded to group allocation.

#### 2.7.1. HE Staining

Heart tissues were fixed in 4% paraformaldehyde for 24–48 h and embedded in paraffin. Sections (5 μm) were stained with HE to assess cardiomyocyte morphology, including cardiomyocyte arrangement, integrity, and the degree of inflammatory cell infiltration in the infarcted and peri‐infarcted regions, as well as the extent of myocardial necrosis.

#### 2.7.2. Masson’s Trichrome Stain

Fibrosis in peri‐infarct myocardium was assessed by Masson’s trichrome staining (Sigma HT15). Collagen fibers appeared blue, cardiomyocytes red, and nuclei black. Fibrosis was quantified as the percentage of collagen‐positive area from three representative fields per section, which served as a direct objective indicator of interstitial fibrosis and scar formation in the myocardium.

#### 2.7.3. WGA Staining

Cardiomyocyte membranes were labeled with Alexa Fluor 488‐conjugated WGA (5–10 μg/mL in PBS). Staining was performed on frozen heart sections (8–10 μm) or dewaxed paraffin sections. The cross‐sectional area of cardiomyocytes in the noninfarcted myocardium was measured under a fluorescence microscope (≥ 50 cells/rat), which was a reliable quantitative index to assess cardiomyocyte hypertrophy and related cardiac structural remodeling.

#### 2.7.4. Immunohistochemical (IHC) Studies

VEGF protein expression in peri‐infarct myocardium was detected by antigen retrieval, incubation with a primary antibody (VEGF, Abcam ab46154, 1:200), and visualization with DAB. Positive staining was quantified.

### 2.8. Real‐Time Quantitative PCR (RT‐qPCR)

Total RNA was isolated from the ischemic area of the left ventricular tissue. Reverse transcription was performed using the Evo M‐MLV RT Kit (AG11706, Accurate Biotechnology, Hunan, China). Quantitative PCR was performed using SYBR Green chemistry (AG11718, Accurate Biotechnology, Hunan, China). Transcript levels of B‐type natriuretic peptide (BNP), choline acetyltransferase (ChAT), VEGF, and α7nAChR were determined, with glyceraldehyde‐3‐phosphate dehydrogenase (GAPDH) as the internal reference gene. Relative gene expression was calculated using the 2^–ΔΔCt^ method. The PCR primer sequences were as follows (Table 1).

**TABLE 1:** Primer sequences for the RT‐qPCR.

Target mRNA	Forward primer	Reverse primer
α7nAChR	ACTATGGCCTCAATCTGCTCATC	CGAAGTATTGTGCTATCAAGGGC
BNP	CAGTCAGTCGCTTGGGCTGT	GCAGAGTCAGAAGCCGGAGT
ChAT	ACACCAATGACCAGCTAAGATTT	GCTTCATACAGAGGGGCTGC
IL‐6	TAGTCCTTCCTACCCCAATTTCC	TTGGTCCTTAGCCACTCCTTC
TNF‐α	AGCCCCCAGTCTGTATCCTT	CTCCCTTTGCAGAACTCAGG
TGF‐β1	GGCTGAACCAAGGAGACGGAA	AGGAGCAGGAAGGGTCGGT
VEGFA	CAGAGCGGAGAAAGCATTTGTT	GTCACATCTGCAAGTACGTTCG
GAPDH	GGCACAGTCAAGGCTGAGAATG	ATGGTGGTGAAGACGCCAGTA

### 2.9. Western Blot

Left ventricular tissues were homogenized in RIPA buffer (Shanghai Biyuntian Biological Co., Ltd., China) supplemented with protease and phosphatase inhibitors. Protein concentrations were determined by BCA assay (Shanghai Epizyme Biomedical Technology Co., Ltd., China). Equal amounts of protein (30 μg/lane) were separated by SDS‐PAGE (Shanghai Epizyme Biomedical Technology Co., Ltd., China) and transferred to PVDF membranes (Beijing Solarbio Science & Technology Co., Ltd., China). Membranes were probed with primary antibodies against ChAT (1:1000, Proteintech, IL, USA), α7nAChR (1:2000, Sangon Biotech (Shanghai) Co., Ltd., China), total Akt (1:1000, HuaBio, China), *p*‐Akt (1:800, HuaBio, China), HIF‐1α (1:1500, HuaBio, China), and VEGF (1:1500, Abclonal, China), followed by HRP‐conjugated secondary antibodies. Bands were visualized by enhanced chemiluminescence, and densitometric analysis was performed using ImageJ. Protein expression was normalized to GAPDH (1:5000, Sangon Biotech (Shanghai) Co., Ltd., China).

### 2.10. Statistical Analysis

Statistical analyses were performed using IBM SPSS Statistics Version 26.0 (IBM Corp., Armonk, NY, USA). Continuous data were expressed as mean ± standard deviation (SD). The assumption of normality was verified using the Shapiro–Wilk test, and homogeneity of variances was assessed using Levene’s test. Intergroup comparisons were performed using one‐way analysis of variance (ANOVA). If the assumption of homogeneity of variance was met, the least significant difference (LSD) post hoc test was used for multiple comparisons. In cases where variances were unequal, the Games–Howell post hoc test was applied. A *p* value of less than 0.05 was considered statistically significant.

## 3. Results

### 3.1. EA Improves Cardiac Function in HF Following MI

**FIGURE 1:**
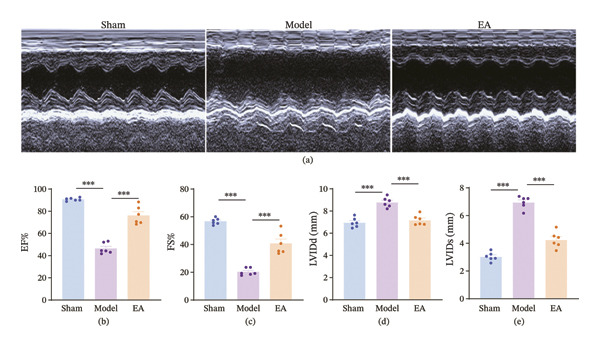
Effects of EA at PC6 on cardiac function in rats with heart failure following myocardial infarction. (a) Echocardiogram of rats after 2 weeks of EA treatment. (b) Left ventricular ejection fraction (EF). (c) Fractional shortening (FS). (d) Left ventricular internal diameter at end‐diastole (LVIDd). (e) Left ventricular internal diameter at end‐systole (LVIDs). ^∗∗∗^ *p* < 0.001 vs. Model.

To evaluate whether EA can improve cardiac dysfunction in rats with HF following MI, echocardiographic assessment was performed after 2 weeks of EA treatment. In the model group, pathological alterations were evident, including left ventricular chamber dilation, weakened or delayed anterior wall motion, paradoxical motion, hyperechoic infarcted regions, and ventricular wall thinning, all indicative of severe cardiac dysfunction compared with the sham group (all *p* < 0.001). In contrast, EA intervention significantly improved cardiac structure and function. EA reduced LV chamber dimensions and increased ventricular wall thickness (Figure 1a). Functional indices also improved: EF and FS were markedly elevated compared with the model group (*p* < 0.001) (Figure 1b, c). Moreover, LV end‐diastolic and end‐systolic diameters (LVIDd and LVIDs) were significantly decreased (*p* < 0.001) (Figure 1d, e). These findings demonstrated that EA enhanced myocardial contractility and cardiac pump performance, thereby improving systemic circulation and mitigating myocardial ischemia in post‐MI HF rats.

### 3.2. EA Regulates ANS Disorders in HF Following MI

**FIGURE 2:**
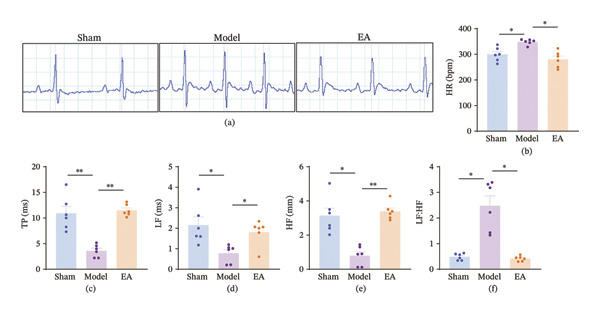
Effects of EA at PC6 on heart rate and autonomic function in rats with heart failure following myocardial infarction (a) Representative ECG. (b) Heart rate (HR). (c) Total power (TP). (d) Low‐frequency power (LF). (e) High‐frequency power (HF). (f) LF/HF ratio. ^∗^ *p* < 0.05, ^∗∗^ *p* < 0.01 vs. Model.

To assess the regulatory effects of EA at PC6 on the ANS function, HRV was analyzed. Compared with the sham group, the model group showed significantly shortened R‐R intervals and elevated heart rate (*p* < 0.05). EA intervention effectively prolonged R‐R intervals (Figure 2a) and reduced heart rate (Figure 2b). Detailed HRV analysis revealed that total power (TP), LF, and HF power components were significantly reduced in the model group (*p* < 0.05), accompanied by an increased LF/HF ratio (*p* < 0.05), indicating a sympathovagal imbalance (Figure 2c–f). In contrast, EA treatment significantly increased TP, LF, and HF levels (*p* < 0.05) while decreasing the LF/HF ratio (*p* < 0.05) relative to the model group (Figure 2c–f). These findings suggested that EA restores autonomic nervous balance by enhancing vagal modulation and suppressing sympathetic overactivity, thereby improving arrhythmic risk in post‐MI HF rats.

### 3.3. EA Improves Cardiac Structure and Attenuates Fibrosis in HF Following MI

**FIGURE 3:**
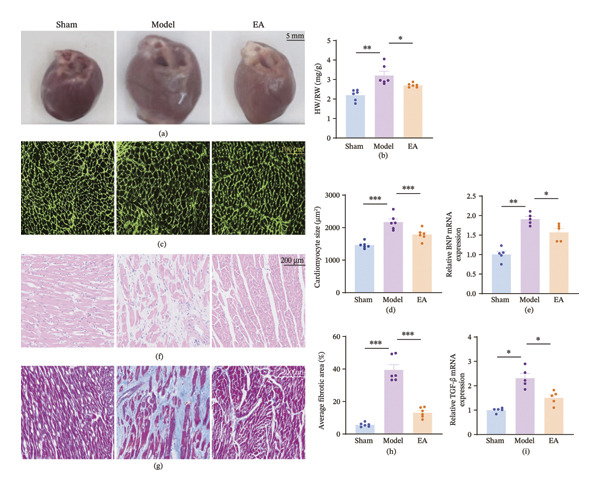
Effects of EA at PC6 on myocardial morphology and molecular markers in rats with heart failure following myocardial infarction. (a) Gross heart morphology (scale bar = 5 mm). (b) Cardiac mass index. (c) WGA fluorescence staining (scale bar = 100 μm). (d) Quantitation of cardiomyocyte cross‐sectional area. (e) BNP mRNA expression. (f) HE staining (scale bar = 200 μm). (g) Masson’s trichrome staining (scale bar = 200 μm). (h) Quantification of fibrotic area. (i) TGF‐β1 mRNA expression. ^∗^ *p* < 0.05, ^∗∗^ *p* < 0.01, ^∗∗∗^ *p* < 0.001 vs. Model.

Cardiac morphometric and molecular analyses revealed significant pathological remodeling in the model group compared with sham controls. The model group showed an increased cardiac mass index (*p* < 0.01), enlarged cardiomyocyte cross‐sectional areas (*p* < 0.001), and upregulated expression of the HF biomarker brain natriuretic peptide (BNP) (*p* < 0.01) (Figure 3a–e). EA intervention effectively reversed these changes by reducing cardiac mass index (*p* < 0.05), decreasing cardiomyocyte size (*p* < 0.001), and suppressing BNP expression (*p* < 0.05) (Figure 3a–e). Histopathological examination further revealed extensive myocardial necrosis, inflammatory infiltration, and sarcolemmal disruption in the model group, whereas EA markedly attenuated these lesions (Figure 3f). Masson’s trichrome staining confirmed pronounced interstitial fibrosis and collagen deposition area in the model group (*p* < 0.001), accompanied by elevated mRNA expression of the profibrotic mediator TGF‐β1 (*p* < 0.05) (Figure 3g–i). Notably, EA treatment significantly reduced collagen accumulation (*p* < 0.001) and downregulated TGF‐β1 expression (*p* < 0.05) (Figure 3g–i). Collectively, these findings indicated that EA at PC6 exerts cardioprotective effects by attenuating pathological hypertrophy, suppressing inflammatory injury and TGF‐β1‐mediated fibrotic remodeling, thereby improving structural and molecular hallmarks of HF.

### 3.4. EA Activates the Vagal–Cholinergic Pathway in HF Following MI

**FIGURE 4:**
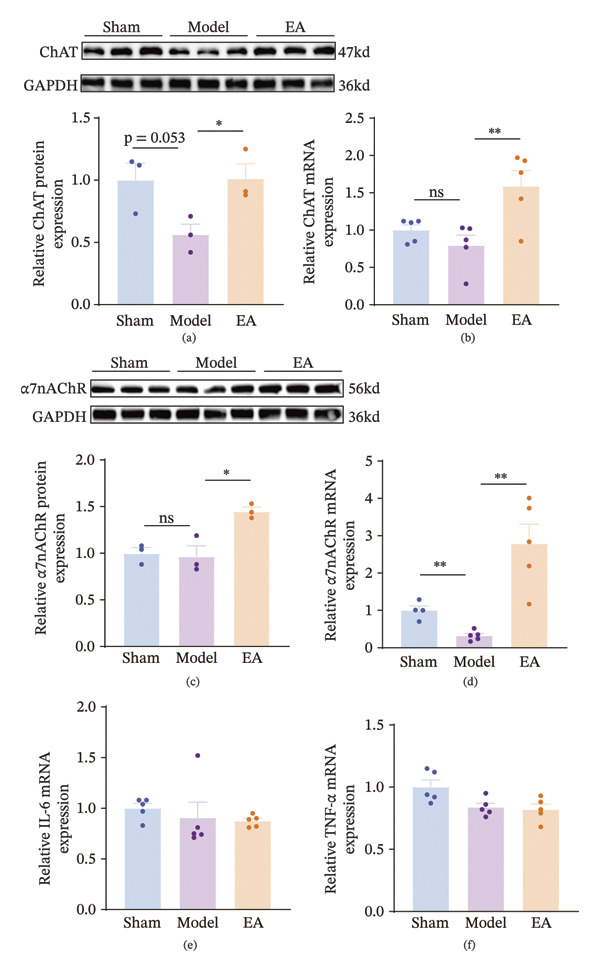
Effects of EA at PC6 on ChAT and α7nAChR expression in rats with heart failure following myocardial infarction. (a) ChAT protein expression. (b) ChAT mRNA expression. (c) α7nAChR protein expression. (d) α7nAChR mRNA expression. (e) IL‐6 mRNA expression. (f) TNF‐α mRNA expression. ^∗^ *p* < 0.05, ^∗∗^ *p* < 0.01 vs. Model.

Building on the observed cardioprotective effects of EA in improving cardiac function, suppressing hypertrophy, and attenuating fibrosis post‐MI, we next explored its underlying mechanisms. RT‐qPCR and Western blot analyses showed that ChAT mRNA and protein expression exhibited a nonsignificant decreasing trend in the model group compared with the sham group (*p* = 0.053) (Figure 4a, b). Notably, EA treatment significantly upregulated ChAT expression at both transcriptional and translational levels (*p* < 0.05) (Figure 4a, b). These results suggested that EA‐induced vagal excitation may enhance acetylcholine (ACh) synthesis by promoting ChAT transcription through transcriptional regulators. In parallel, the model group showed a marked downregulation in α7nAChR protein expression (*p* < 0.01) (Figure 4c, d). EA treatment effectively reversed this decline, significantly elevating α7nAChR expression at both mRNA and protein levels (*p* < 0.05) (Figure 4c, d). Interleukin‐6 (IL‐6) and tumor necrosis factor‐α (TNF‐α), as key proinflammatory factors during the acute phase of MI [27], had returned to baseline by postoperative Day 21 across all groups (Figure 4e, f), indicating resolution of the acute inflammatory response. These results demonstrate that EA at PC6 persistently activates vagal–cholinergic signaling, enabling its continued functional role during the late remodeling phase.

### 3.5. EA Upregulates the Cholinergic Pathway‐Mediated Akt/HIF‐1α/VEGF Pro‐Angiogenic Signaling Axis

**FIGURE 5:**
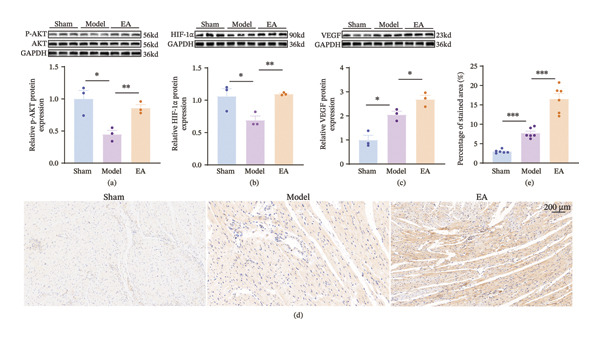
Effects of EA at PC6 on the expression of Akt/HIF‐1α/VEGF in the myocardium of rats with heart failure following myocardial infarction. (a) Protein expression levels of p‐Akt and Akt. (b) Protein expression levels of HIF‐1α. (c) Protein expression levels of VEGF. (d) and (e) VEGF immunohistochemistry and quantitative analysis. ^∗^ *p* < 0.05, ^∗∗^ *p* < 0.01, ^∗∗∗^ *p* < 0.001 vs. Model.

As a pivotal downstream effector of α7nAChR signaling, Akt plays a central role in regulating cell survival, proliferation, and apoptosis suppression in cardiovascular injury [28, 29]. Western blot analysis demonstrated that p‐Akt was significantly reduced in the model group compared with the sham group (*p* < 0.05), indicating impaired survival signaling. EA intervention markedly restored p‐Akt expression (*p* < 0.01) (Figure 5a). Hypoxia‐inducible factor‐1α (HIF‐1α), a key regulator of ischemic adaptation and pro‐angiogenic signaling, was also significantly downregulated in the model group (*p* < 0.05). EA treatment effectively rescued HIF‐1α expression (*p* < 0.01) (Figure 5b). During myocardial ischemia, Akt and HIF‐1α act synergistically to regulate cardioprotection and angiogenic signaling pathways [30]. Compared with the sham group, the model group exhibited a paradoxical increase in VEGF protein expression (*p* < 0.05), and EA further enhanced this response (*p* < 0.05) (Figure 5c). Immunohistochemistry revealed markedly increased VEGF immunoreactivity in cardiomyocytes of the model group (*p* < 0.001), which was further intensified following EA treatment (*p* < 0.001) (Figure 5d and e). Together, these findings indicated that EA activates the α7nAChR‐dependent survival cascade and enhances Akt/HIF‐1α‐VEGF‐associated pro‐angiogenic signaling in the ischemic myocardium.

## 4. Discussion

Cardiac remodeling following acute myocardial infarction (AMI) is a temporally staged process. The initial necroinflammatory phase, characterized by leukocyte infiltration and clearance of necrotic debris, occurs within 1–3 days [31], followed by fibroblast and endothelial cell proliferation between 3 and 7 days [32]. Remodeling then enters an early transition stage at 1–2 weeks, progressing to scar maturation and geometric remodeling at 2–4 weeks, and ultimately evolving into a chronic stabilization phase at 4–8 weeks [33]. Importantly, most previous studies investigating acupuncture or neuromodulation in MI have primarily focused on the acute inflammatory phase or on isolated functional endpoints. In contrast, the present study specifically targets the subacute reparative phase (Days 7–21), a critical but underexplored window characterized by active vascular remodeling and structural repair. The timing avoided interference from the acute necroinflammatory phase while maximizing sensitivity to detect pro‐angiogenic reparative responses [34]. Moreover, by integrating autonomic regulation (HRV indices) with structural remodeling markers and molecular signaling along the α7nAChR‐Akt‐HIF‐1α‐VEGF axis, our study provides a multilevel framework linking neuromodulation to myocardial repair processes.

Our findings are consistent with prior reports that EA at PC6 improves cardiac function and suppresses myocardial fibrosis in ischemic heart disease models. Echocardiographic assessment confirmed preserved LVEF and FS, along with reduced LVIDd and LVIDs, indicating preserved myocardial contractility and suppression of ventricular dilation [35, 36]. These results corroborate earlier studies demonstrating acupuncture’s beneficial effects on cardiac function post‐MI. Moreover, HRV analysis showed decreased heart rate and prolonged R–R intervals, reflecting enhanced vagal tone and reduced sympathetic overactivation. The increases in TP, LF, and HF components, along with a reduced LF/HF ratio, suggested vagal dominance and autonomic balance [21, 37, 38]. HF power is predominantly associated with parasympathetic activity, whereas the LF/HF ratio serves as an integrative index of sympathovagal balance rather than a direct measure of sympathetic tone. The normalization of these HRV parameters therefore supports the interpretation that EA may restore autonomic equilibrium. Given the established association between autonomic imbalance and adverse prognosis in HF, these findings may have potential implications for cardiac stability and clinical outcomes, although direct effects on clinical events were not evaluated in the present study. In addition to functional improvements, EA suppressed maladaptive structural remodeling. BNP expression and cardiac mass index were reduced, consistent with attenuation of pathological hypertrophy [39, 40]. Histological analyses revealed mitigation of necrosis, inflammatory infiltration, and collagen deposition, accompanied by decreased TGF‐β1 expression, suggesting inhibition of profibrotic signaling [41]. These findings indicated that EA may exert multimodal cardioprotective effects, potentially involving the simultaneous attenuation of hypertrophy, inflammation, and fibrosis.

ChAT is the rate‐limiting enzyme for ACh synthesis, whereas α7nAChR is a key receptor mediating the CAP [42, 43]. Notably, during the remodeling phase following MI, differential patterns emerged between classical proinflammatory cytokines and cholinergic signaling components. By postoperative Day 21, IL‐6 and TNF‐α mRNA levels showed no significant intergroup differences, consistent with resolution of the acute inflammatory responses [44]. In contrast, the model group exhibited reduced ChAT expression, indicative of impaired cholinergic capacity following cardiac injury. Moreover, while α7nAChR expression did not differ significantly between sham and model groups, EA treatment markedly increased its expression. This finding suggested that EA at PC6 may enhance cholinergic pathway activity even after systemic inflammation has subsided [45]. Beyond its established role in suppressing cytokine release during the acute phase, α7nAChR is increasingly recognized as a regulator of prosurvival, pro‐angiogenic, and antifibrotic pathways [46, 47]. Thus, EA‐induced upregulation of α7nAChR may contribute to the activation of protective signaling cascades such as the PI3K/Akt pathway [48], thereby extending cholinergic benefits from inflammation control to structural remodeling. Importantly, the coordinated upregulation of ChAT and α7nAChR observed at this late remodeling stage suggests a sustained enhancement of the cardiac cholinergic pathway rather than a transient response to acute inflammation. This pathway‐level modulation is temporally aligned with improved autonomic indices and cardiac function, supporting a working model whereby EA may promote long‐term vagal–cardiac cholinergic signaling capacity during post‐MI remodeling.

In our model, MI induced a reduction in Akt and HIF‐1α protein levels at the chronic remodeling phase, consistent with impaired prosurvival and hypoxic‐response signaling in the injured myocardium [49, 50]. The concomitant upregulation of VEGF in the model group may represent a localized ischemia‐induced compensatory response, occurring despite overall suppression of Akt/HIF‐1α signaling. EA restored Akt and HIF‐1α expression, enhanced VEGF expression in viable myocardium, and thereby amplified pro‐angiogenic signaling beyond the model’s endogenous response. These results align with the established roles of Akt in cell survival, HIF‐1α in hypoxia adaptation, and VEGF in neovascularization [51, 52]. These observations suggest that EA may restore intracellular survival and hypoxic signaling (Akt/HIF‐1α) while simultaneously augmenting VEGF‐associated pro‐angiogenic signaling, providing a potential mechanistic basis for improving capillary formation, reducing fibrosis, and preserving function. We acknowledge, however, that VEGF upregulation alone does not directly confirm functional angiogenesis; future studies assessing capillary density (e.g., CD31/α‐SMA staining) and myocardial perfusion are warranted to validate whether these molecular changes translate into true neovascularization. Importantly, this pattern suggests that EA did not merely enhance VEGF expression, but rather restored the coupling between intracellular survival signaling (Akt/HIF‐1α) and downstream angiogenic signaling pathways, thereby potentially facilitating a myocardial environment more permissive for vascular regeneration during chronic post‐MI remodeling. Although the present findings were obtained in a rodent model, the autonomic dysregulation, maladaptive remodeling, and impaired angiogenic repair targeted by EA represent fundamental processes shared by human post‐MI HF. These observations therefore support EA as a potential adjunctive strategy that warrants further clinical investigation.

While this study delineates the sequential activation of an EA‐mediated vagal cholinergic–α7nAChR–Akt–HIF‐1α–VEGF axis and its association with improved cardiac function, several limitations should be acknowledged. First, the proposed mediating role of the vagal cholinergic pathway and α7nAChR signaling should be considered a mechanistic hypothesis supported by correlative evidence rather than definitive causal proof. Although the sample size is consistent with exploratory mechanistic studies, it may limit statistical power and increase the risk of Type II errors for certain endpoints, particularly those showing trend‐level changes (e.g., selected molecular markers). Accordingly, negative or marginal findings, such as inflammatory cytokine expression, should be interpreted with caution. To establish causality, future studies should expand cohort sizes and adopt targeted loss‐of‐function strategies, including pharmacological blockade of α7nAChR (e.g., methyllycaconitine), Akt inhibition, vagal denervation, and genetic loss‐of‐function models. These approaches will be critical to determine the necessity of α7nAChR signaling in mediating EA‐induced cardioprotection. Second, although VEGF upregulation was observed, functional angiogenesis and myocardial perfusion were not directly quantified. Subsequent work should assess capillary density using endothelial markers (e.g., CD31 and α‐SMA) and apply perfusion‐based imaging techniques to confirm the functional relevance of EA‐induced angiogenic signaling. Finally, as the findings were derived from a single experimental model, validation across additional HF models and complementary *in vitro* studies using primary cardiomyocytes and cardiac microvascular endothelial cells will be necessary to strengthen generalizability and translational relevance.

In summary, this study provides an integrative analysis across autonomic, structural, and molecular levels, suggesting that EA may shift the postischemic myocardium from a state of impaired survival signaling toward an environment permissive for pro‐angiogenic signaling and structural repair. Mechanistically, our data suggest that EA may engage vagal cholinergic signaling, enhance ChAT and α7nAChR expression, and involve the Akt/HIF‐1α/VEGF axis, thereby supporting a working model linking neuromodulation to intracellular hypoxic adaptation and vascular remodeling. From a translational perspective, these findings extend the neuromodulation field by providing a mechanistic framework rather than isolated functional observations. EA therefore represents a potential nonpharmacological intervention with the potential to complement standard care, although its clinical efficacy remains to be established. Future studies should focus on causal validation, direct assessment of functional angiogenesis, and well‐designed clinical trials to determine whether these preclinical findings translate into therapeutic benefit in patients with HF.

**Nomenclature**
α7nAChR: α7‐nicotinic acetylcholine receptor
ACh: Acetylcholine
Akt: Protein kinase B
ANS: Autonomic nervous system
BNP: Brain natriuretic peptide
CAP: Cholinergic anti‐inflammatory pathway
ChAT: Choline acetyltransferase
EA: Electroacupuncture
ECG: Electrocardiogram
EF: Ejection fraction
FS: Fractional shortening
HF: Heart failure
HIF‐1α: Hypoxia‐inducible factor‐1α
HRV: Heart rate variability
IL‐6: Interleukin‐6
LAD: Left anterior descending artery
LVIDd: Left ventricular internal diameter at end‐diastole
LVIDs: Left ventricular internal diameter at end‐systole
MI: Myocardial infarction
PC6: Neiguan acupoint
TGF‐β1: Transforming growth factor‐β1
TNF‐α: Tumor necrosis factor‐α
TP: Total power
VEGF: Vascular endothelial growth factor
VNS: Vagus nerve stimulation
WGA: Wheat germ agglutinin

## Author Contributions

Jia Zhou, Aijun Liu, and Ke Wang contributed to the study conception and design. Jia Zhou, Aijun Liu, and Ke Wang supervised the experiments. Weiting Wang and Jiaojiao Wu performed model preparation, data collection, and analysis. Yueling Xu and Qiao Lin contributed to model preparation and data collection. Weiting Wang wrote the initial draft of the manuscript. All authors commented on previous versions of the manuscript.

## Funding

This study was supported by grants to Jia Zhou from the Science and Technology Commission of Shanghai Municipality (25Y12800700, 20MC1920500), the National Natural Science Foundation of China (82074163), the Three Year Action Plan for Shanghai to Further Accelerate the Inheritance, Innovation and Development of Traditional Chinese Medicine (2025–2027) (1‐1‐2), and the Key Construction Discipline of National Administration of Traditional Chinese Medicine (ZYYZDXK‐2023068).

## Disclosure

All authors have reviewed and approved the final manuscript.

## Ethics Statement

All experimental animals in this study were humanely cared for in accordance with the Guidelines for the Health and Care of Laboratory Animals at Yueyang Hospital of Integrated Traditional Chinese and Western Medicine, Shanghai University of Traditional Chinese Medicine (hereinafter referred to as Yueyang Hospital). All experiments were approved by the Animal Ethics Committee of Yueyang Hospital (approval number: YYLAC‐2023‐220‐1).

## Conflicts of Interest

The authors declare no conflicts of interest.

## Data Availability

The data used to support the findings of this study are included within the article.
